# The effect of biogeographic and phylogeographic barriers on gene flow in the brown smoothhound shark, *Mustelus henlei*, in the northeastern Pacific

**DOI:** 10.1002/ece3.1458

**Published:** 2015-03-17

**Authors:** Chris L Chabot, Mario Espinoza, Ismael Mascareñas-Osorio, Axayácatl Rocha-Olivares

**Affiliations:** 1 Department of Biology, California State University, NorthridgeNorthridge, California, 91330; 2 Unidad de Investigación Pesquera y Acuicultura (UNIP), Centro de Investigación en Ciencias del Mar y Limnología (CIMAR), Universidad de Costa Rica11501–2060, San José, Costa Rica; 3 Centro para la Biodiversidad Marina y la Conservación A.C.23090, La Paz, B.S.S, México; 4 Molecular Ecology Laboratory, Department of Biological Oceanography, CICESECarretera Ensenada-Tijuana 3918, Ensenada, Baja California, 22860, México

**Keywords:** Microsatellite, mitochondria, phylogeography, shark, Triakidae

## Abstract

We assessed the effects of the prominent biogeographic (Point Conception and the Peninsula of Baja California) and phylogeographic barriers (Los Angeles Region) of the northeastern Pacific on the population connectivity of the brown smoothhound shark, *Mustelus henlei* (Triakidae). Data from the mitochondrial control region and six nuclear microsatellite loci revealed significant population structure among three populations: northern (San Francisco), central (Santa Barbara, Santa Catalina, Punta Lobos, and San Felipe), and southern (Costa Rica). Patterns of long-term and contemporary migration were incongruent, with long-term migration being asymmetric and occurring in a north to south direction and a lack of significant contemporary migration observed between localities with the exception of Punta Lobos that contributed migrants to all localities within the central population. Our findings indicate that Point Conception may be restricting gene flow between the northern and central populations whereas barriers to gene flow within the central population would seem to be ineffective; additionally, a contemporary expansion of tropical *M. henlei* into subtropical and temperate waters may have been observed.

## Introduction

Biogeographic and phylogeographic barriers are known to disrupt population connectivity and are produced by currents, changes in sea surface temperature (SST), physical barriers, upwelling, and resource availability (Palumbi 1994; Dawson 2001; Dawson et al. 2001; Jacobs et al. 2004). The eastern Pacific is an area comprising numerous biogeographic and phylogeographic barriers, the most prominent being located at Cape Mendocino, Point Conception, the Los Angeles Region (LAR), Punta Eugenia, the Peninsula of Baja California, the Sinaloan Gap, the Central American Gap, the Isthmus of Panama, and the Equator (Rawson et al. 1999; Stepien et al. 2000; Dawson 2001; Dawson et al. 2001, 2006; Jacobs et al. 2004; Robertson and Cramer 2009). The impact of these barriers on population connectivity can vary in magnitude depending on their age and degree of obstruction. For example, the Isthmus of Panama, which is relatively recent (∼3.5–3.1 million years ago), has altered current patterns, increased tidal amplitudes, affected upwelling, and severed gene flow between eastern Pacific, western Atlantic, and Caribbean taxa (Coates and Obando 1996; Dick et al. 2003; Coates et al. 2004; Duncan et al. 2006; Keeney and Heist 2006). Barriers can also produce population structure along latitudinal gradients. Chabot and Allen (2009) have demonstrated significant population structure among populations of the soupfin shark, *Galeorhinus galeus*, straddling the Equator, hypothesizing that the species affinity for cool temperate waters limits its ability to cross-warm equatorial waters.

**Figure 1:**
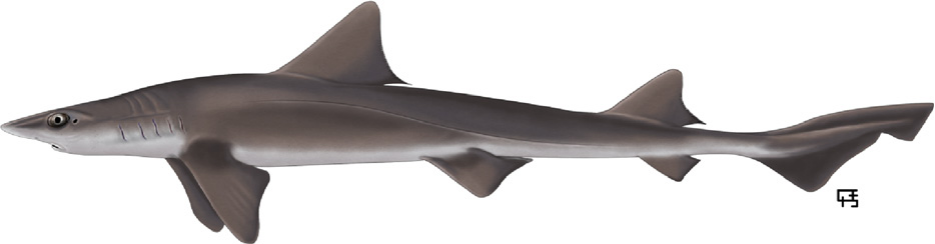
*Mustelus henlei*.

Within the eastern Pacific, the brown smoothhound shark, *Mustelus henlei* (Gill, 1863) (Triakidae) (Fig.1), has the greatest distribution of any *Mustelus* within the region. The species is commonly encountered in temperate to tropical coastal waters at depths ranging from the shallow intertidal to 200 m (Ebert 2003) with a geographic range spanning Coos Bay, Oregon (North America) to Peru and Ecuador (South America) (Compagno 1984; Eschmeyer et al. 1999) and a recent discovery of the species off the coast of Washington (North America) (D. Ebert, pers. comm.). Although *M. henlei* is considered to occur primarily in the northeastern Pacific, observations of this species off the coasts of Peru and Ecuador (Compagno 1984) may indicate the ability of this species to cross-warm equatorial waters. Estimates of maximum total length and age are reported as 100 cm and 13 years (Yudin and Cailliet 1990; Smith et al. 1998) with estimates of age at first maturity occurring between 2 and 3 years (Yudin and Cailliet 1990). Reproduction is through placental viviparity with periods of gestation lasting ∼1 year and each female capable of producing between 1 and 20 pups depending on geographic locality (*n* = 1–10 in Central California vs. 1–21 in the northern Gulf of California) (Yudin 1987; Pérez Jiménez and Sosa Nishizaki 2008). During the winter, *M. henlei* from San Francisco Bay are known to migrate out of the Bay when salinity decreases due to increased rainfall and have been observed offshore from November to April (Compagno 1984; Yudin and Cailliet 1990). However, it is currently unknown where these sharks migrate to or the extent of their migration.

In order to determine the impact of eastern Pacific biogeographic and phylogeographic barriers on gene flow within the Triakidae, both nuclear (microsatellite) and mitochondrial (control region) genetic markers were used to reconstruct the phylogeographic history of *M. henlei* as well as to elucidate contemporary gene flow within the distribution of the species. The matrilineally transmitted, noncoding, mitochondrial control region (mtCR) is considered a selectively neutral marker that lacks recombination and mutates at a relatively high rate, making it effective for detecting historic or evolutionary structure among populations (Avise 2004; Wang 2010). Due to their relatively high mutation rates and bi-parental mode of transmission, microsatellites are also ideal for testing gene flow among populations and provide a more contemporary glimpse into the gene flow of species (Avise 2004; Wang 2010). The purpose of this study was to determine the patterns of population connectivity among sample localities of *M. henlei* throughout its northeastern Pacific distribution and to identify biogeographic and phylogeographic barriers that may be responsible for shaping these patterns.

## Materials and Methods

### Sample acquisition and DNA extraction

Adult specimens of both male and female *M. henlei* were collected from throughout the species' distribution by trawling, beach seine, and gill netting. An adult *M. henlei* from 2012 was deposited at the Scripps Institute of Oceanography as a voucher specimen (SIO 13–25). In total, 169 tissue samples were collected from six geographic locations: 31 from San Francisco Bay, CA (37.7750° N, 122.4183° W), 34 from Santa Barbara, CA (34.4208° N, 119.6972° W), 30 from Santa Catalina Island, CA (33.3749° N, 118.4199° W), 30 from Punta Lobos, Baja California Sur, Mexico (26.9841667° N, 113.9994° W), 12 from the Upper Gulf of California (G.O.C.) at San Felipe, Baja California, Mexico (31.0275° N, 114.8353° W) and 32 from Costa Rica (9.9500° N, 84.0000° W). Fin clips (1 cm^2^) were taken from the first dorsal fin, placed in 95% ethanol, and stored at −20°C at UCLA for long-term storage until DNA could be extracted. DNA was extracted using the DNeasy Blood and Tissue Kit (Qiagen, Valencia, CA) following the manufacturer's protocols.

### Mitochondrial DNA (mtDNA) sequencing

The mitochondrial control region was amplified as described in Chabot and Allen (2009) using universal shark mitochondrial control region primers. BigDye 3.1 (Life Technologies, New York, NY) dye-termination sequencing was carried out using sequencing primers designed for *M. henlei*: MhenFSeqPrimer 5′- TGC TAC GAC GCG CAA AAG CC and MhenRSeqPrimer 5′-CGT CGG CCC TCG TTT TAG GGG. Sequencing reactions were performed in an Applied Biosystems GeneAmp 9700 thermocycler (Life Technologies) for 35 cycles of 90°C for 10 sec, 50°C for 10 sec, and 60°C for 4 min, followed by direct sequencing in an Applied Biosystems 3130X Genetic Analyzer (Life Technologies). Sequencing products were validated by eye in GENEIOUS PRO 5.1.4 (Biomatters Ltd., Auckland, New Zealand) and aligned using CLUSTALW (Thompson et al. 1994) within GENEIOUS PRO.

### mtDNA genetic diversity and female-mediated gene flow

ARLEQUIN 3.5.1.2 (Excoffier and Lischer 2010) was used to estimate haplotype diversity (*h*), mean nucleotide diversity (*π*), mean pairwise difference, and the population mutation parameter *θ*_s_ (based on the number of segregating sites, sample size, and *θ* for a sample of non-recombining DNA). Effective female population size, *N*_*ef*_, was estimated for each sample locality and based on the equation *θ*_s_ = 2*N*_*ef*_u (*u* = 2 *μ*k, where *μ* is the mutation rate and k is the number of nucleotides). The mutation rate, *μ*, of 0.0067 sequence divergence per million years based on lemon sharks, *Negaprion* (Schultz et al. 2008), was used in all calculations of *N*_*ef*_. An analysis of molecular variance (AMOVA) (Excoffier et al. 1992) was performed in GENALEX 6.501 (Peakall and Smouse 2006) to calculate the divergence estimator Φ_ST_ for all sample localities and all estimates were tested nonparametrically (9999 bootstrapped replicates). Pairwise estimates of Φ_ST_ were generated for all pairs of sample localities in GENALEX, and significance was tested via permutation. Significance was adjusted to correct for multiple tests using the sequential Bonferroni correction of Rice (1989). Spatial autocorrelation was tested using an isolation by distance model in GENALEX and statistical significance was determined by permutation (9999 replicates). As the Costa Rican sample locality has the greatest geographic distance from all localities due to a lack of sampling along the Mexican coast between Costa Rica and the northern localities, a significant pattern of isolation by distance may be observed solely due to a lack of sampling along the Mexican coast. To account for this, analyses were performed both with and without Costa Rica. Coalescent-based estimates of long-term gene flow among the six sample localities were generated in MIGRATE-N 3.2.7 (Beerli and Felsenstein 2001) and based on *M* (where *M *= *m/μ* and *m* is the migration rate per generation and *μ* is the mutation rate). For the MIGRATE-N analysis, an initial analysis under the default parameters was used to estimate *θ* for all populations as well as migration rates for all population pairwise comparisons. Following this, two subsequent runs with different starting seeds using these estimates as priors were performed and averaged to generate *θ* and *M*. A median-joining network of haplotypes was constructed in NETWORK 4.6.1 (Bandelt et al. 1999; www.fluxus-engineering.com) to visualize haplotype clustering and diversity. Following the generation of an initial median-joining network, the MP option (Polzin and Daneshmand 2003) was used to calculate and screen the network to delete superfluous median vectors and links that are not contained in the shortest trees.

### Female population expansion

Tajima's *D* (Tajima 1989, 1993, 1996) and Fu's *F*_S_ (Fu 1997) were calculated in ARLEQUIN and used to detect historic demographic expansions. Tajima's *D* compares two estimators of the population parameter *θ* (*θ*_S_ based on the number of segregating sites and *θ*_*π*_ based on the mean number of pairwise differences between haplotypes) (Tajima 1989, 1996). Under the infinite sites model, both estimates should be equal indicating a population at equilibrium and a *D* of ∼0. In contrast, significantly negative values of *D* (*P *≤* *0.05) are indicative of populations not in equilibrium due possibly to a recent range expansion or recovery from a population bottleneck (Tajima 1989, 1993, 1996; Aris-Brosou and Excoffier 1996). Similar to Tajima's *D*, Fu's *F*_S_ statistic is also sensitive to population expansion under the infinite sites model with significantly negative *F*_S_ values (*P *≤* *0.02) indicating an excess of novel haplotypes and a departure from equilibrium (Fu 1997). Population expansion times were estimated from Tau (*τ*) values, the amount of mutational time in which all lineages within a sample coalesce, derived from mismatch distributions (Rogers and Harpending 1992) calculated in ARLEQUIN. Generations since divergence, *t*, were estimated by the equation *τ* = 2*μt* (with *t *= the number of generations and *μ *= the mutation rate) (Rogers and Harpending 1992). Expansion time, T, was estimated by multiplying *t* by 4.7, the average generation time of *M. henlei* (Cortes 2002).

### Microsatellite genotyping and analyses

Six nuclear microsatellite loci from Chabot (2012) (Mh1, Mh6, Mh13, Mh25, Mh34, and Mh36) were used to genotype individuals of *M. henlei* from all sample localities following the procedures of Chabot (2012). MICRO-CHECKER 2.2.3 (van Oosterhout et al. 2004) was used to detect the presence of null alleles, large allele dropout, and stuttering. Departures from Hardy–Weinberg equilibrium (HWE), observed heterozygosity (H_O_), and expected heterozygosity (H_E_) were estimated in GENEPOP 4.0 (Raymond and Rousset 1995; Rousset 2008). FSTAT 2.9.3.2 (Goudet 2003) was used to test linkage disequilibrium (LD), provide the total number of alleles, and estimate allelic richness (A_R_).

### Genetic divergence and population structure

STRUCTURE 2.3.3 (Pritchard et al. 2000; Falush et al. 2003, 2007) was used to estimate population subdivision in *M. henlei*. Number of subpopulations (*K*) was estimated with five independent runs of *K *=* *1–10, each was performed with 10^6^ MCMC repetitions and a burn-in of 10^5^ steps under the admixture model with correlated allele frequencies. The optimal number of subpopulations was estimated using Δ*K* of Evanno et al. (2005) as implemented in STRUCTURE HARVESTER 0.6.94 (Earl and vonHoldt 2012). Overall population structure was estimated by AMOVA as implemented in GENALEX. Estimates were tested nonparametrically (9,999 bootstrapped replicates). Pairwise population estimates of *F*_ST_ were generated for all pairs of sample localities in GENALEX, and significance was tested via permutation. The sequential Bonferroni correction of Rice (1989) was used to correct for multiple testing. Spatial autocorrelation was tested using an isolation by distance model in GENALEX (Peakall and Smouse 2006), and statistical significance was determined by permutation (9999 replicates).

*F*_ST_ is commonly used to assess population subdivision; however, due to the high mutation rate of microsatellites, *F*_ST_ may underestimate population subdivision (Rousset 1996). Therefore, Hedrick′s *G*'_ST_ (Hedrick 2005; Meirmans and Hedrick 2011) and Jost's (2008) were estimated in GENALEX and statistical significance was evaluated by permutation (9999 replicates). Both estimators produce values between 0 and 1 with 0 indicating complete panmixia and 1 being indicative of a lack of migration.

### Biparental gene flow

Historic and contemporary estimates of gene flow were generated in MIGRATE-N and BAYESASS 3.0.3 (Wilson and Rannala 2003). For the MIGRATE-N analysis, an initial analysis under the default parameters was used to estimate *θ* for all populations as well as rates of gene flow for all population pairwise comparisons. Following this, five subsequent runs with different starting seeds using these estimates as priors were performed and averaged to generate *θ* and *M*. For BAYESASS, five analyses were performed with different starting seeds and were averaged to produce a single estimate of migration with 95% credible sets for each pairwise comparison. Each analysis consisted of twenty million iterations with the first two million iterations discarded as burn-in and Δ_*A*_, Δ_*F*_, and Δ_*M*_ set to 0.3, 0.6, and 0.1, respectively. To determine the convergence of runs, mean log-probabilities were compared among runs and total log-likelihood was plotted versus iteration in TRACER 1.5 (Rambaut and Drummond 2007) to determine whether runs consisted of regular oscillations (i.e., no persistent peaks and valleys).

### Population bottleneck

When populations undergo recent declines in effective population sizes (*N*_e_) due to bottlenecks, observed heterozygosity at neutral loci is generally greater than that expected by the number of alleles. To detect recent declines in *N*_e_ within populations of *M. henlei*, BOTTLENECK 1.2.02 (Piry et al. 1999) was used under the two-phase model (TPM) with 20,000 replications, 5% of multistep mutations, and variance among multiple steps of 12 as recommended for microsatellites (Piry et al. 1999). The significance of any observed heterozygote excess was assessed by a one-tailed Wilcoxon's signed rank test; a test considered to be the most informative and robust for microsatellites (Piry et al. 1999). To further investigate the possibility of bottlenecked populations of *M. henlei,* the M ratio test of Garza and Williamson (2001) was calculated for each population in M_P_val GLO.3 (Garza and Williamson 2001) and a critical value of M_C_ was estimated by CRITICAL_M (Garza and Williamson 2001). The M ratio test calculates the ratio of the number of alleles at a given locus and the range of allele sizes with the expectation that the number of alleles in a population that has experienced a bottleneck will be reduced faster than the range of allele sizes (Garza and Williamson 2001). Parameters for the M ratio test *θ*, *p*_s_ (proportion of single-step mutations), and Δ_g_ (average size of non-single-step mutations) were obtained from MIGRATE-N (*θ*) and Garza and Williamson (2001) (*P*_s_ = 0.88 and Δ_g_ = 2.8). Prebottleneck values of *θ* for CRITICAL_M were estimated from equilibrium heterozygosities (H_e_) (Table1B) and the equation 1-H_e_ = 1/1 + *θ*. Conservative values recommended by Garza and Williamson (2001) for *P*_s_ = 0.9 and Δ_*g*_ = 3.5 were used for CRITICAL_M.

**Table 1:** Genetic diversity of *Mustelus henlei*. (A) N, haplotype number; haplotype diversity (*h*); mean nucleotide diversity (*π*); mean pairwise difference (MPD); coancestry coefficient (*θ*_S_); effective female population size (*N*_ef_); ^*^ indicates significant *P* values for Tajima's *D* and Fu's *F* < 0.05 and 0.02, respectively. (B) *N*, number of individuals, H_O_ avg. observed heterozygosity; H_E_ avg. expected heterozygosity; B, Wilcoxon's test probability of heterozygote excess under the two-phase model (*TPM*); GW, avg. Garza–Williamson M ratio (^*^ indicates M ratios less than M_C_); A, number of alleles; AR, avg. allelic richness; PA, private alleles

Locality	*N*	*h*	*π*	MPD	*θ*_S_	*N*_ef_	Tajima's *D*	Fu's *F*
(A) Summary of mtDNA statistics for *Mustelus henlei*.								
Overall	126	0.77 ± 0.04	0.004 ± 0.003	2.94 ± 1.55	4.81	2,45,408	−1.53^*^	−10.34^*^
San Francisco	27	0.48 ± 0.05	0.001 ± 0.001	0.48 ± 0.43	0.26	12,586	1.40	1.51
Santa Barbara	20	0.83 ± 0.08	0.005 ± 0.003	3.41 ± 1.80	3.38	1,72,449	−0.60^*^	−2.23^*^
Santa Catalina	21	0.10 ± 0.80	0.001 ± 0.001	0.86 ± 0.60	1.11	56,633	−1.39^*^	2.59
Punta Lobos	23	0.83 ± 0.07	0.006 ± 0.004	4.27 ± 2.20	3.52	1,79,849	−0.79^*^	−0.93
San Felipe	11	0.80 ± 0.11	0.004 ± 0.003	2.69 ± 1.55	1.71	87,370	0.01	−0.80
Costa Rica	24	0.87 ± 0.05	0.004 ± 0.003	2.26 ± 1.29	2.41	1,50,474	−0.21	−3.31^*^

Locality	*N*	H_O_	H_E_	A	A_R_	PA	B	GW
(B) Summary microsatellite statistics for *Mustelus henlei*.								
Overall	169	0.45	0.56	44	4.11	–	–	–
San Francisco	31	0.45	0.49	28	2.77	4	NS	0.56^*^
Santa Barbara	34	0.45	0.56	35	4.64	5	NS	0.79
Santa Catalina	30	0.47	0.54	26	3.73	0	NS	0.76
Punta Lobos	30	0.45	0.52	28	4.04	0	NS	0.76
San Felipe	12	0.54	0.52	22	3.44	0	NS	0.74
Costa Rica	32	0.40	0.40	22	2.82	3	NS	0.83

## Results

### Mitochondrial data

#### Nucleotide and haplotype diversity

PCR and sequencing were attempted for all 169 samples; however, of these samples, 126 yielded high-quality control region sequences of approximately 700 bp that were used for this study. Within all localities (excluding San Felipe), a minimum of at least 20 individuals were amplified successfully and sequenced. Nucleotide composition of control region sequences was as follows: cytosine, 19.00%, thymine, 35.84%, adenine, 30.14%, and guanine, 15.01%. Overall, 28 polymorphic sites were observed in control region sequences of *M. henlei* consisting of 16 transitions, 11 transversions, and a single insertion/deletion defining 27 haplotypes (GenBank Accession Numbers KC208467–KC208482 and KJ530691–KJ530703). Of the 27 haplotypes, 20 were unique to their sample locality (San Francisco = SF1; Santa Barbara = SB4, SB17, SB28; Punta Lobos = PL1, PL10, PL27, PL30, PL39; San Felipe = GOC11; Costa Rica = CR1, CR2, CR6, CR11, CR16, CR20, CR26, CR38, CR40, CR43). Of the remaining seven haplotypes, SB15 was observed in all localities (excluding Costa Rica) with frequencies ranging from 0.39 to 0.95; SB11, SB14, and PL18 were observed at Santa Barbara, Punta Lobos, and San Felipe, and SB19 was observed at Santa Barbara and Punta Lobos. Although the Costa Rica sample was composed almost entirely of unique haplotypes, haplotype SB14 was observed at this locality as well. Of the sampled localities, only Santa Catalina lacked any unique haplotypes and was dominated by haplotypes SB15 (0.95) and SB23 (0.05).

#### mtDNA divergence and female gene flow

**Figure 2:**
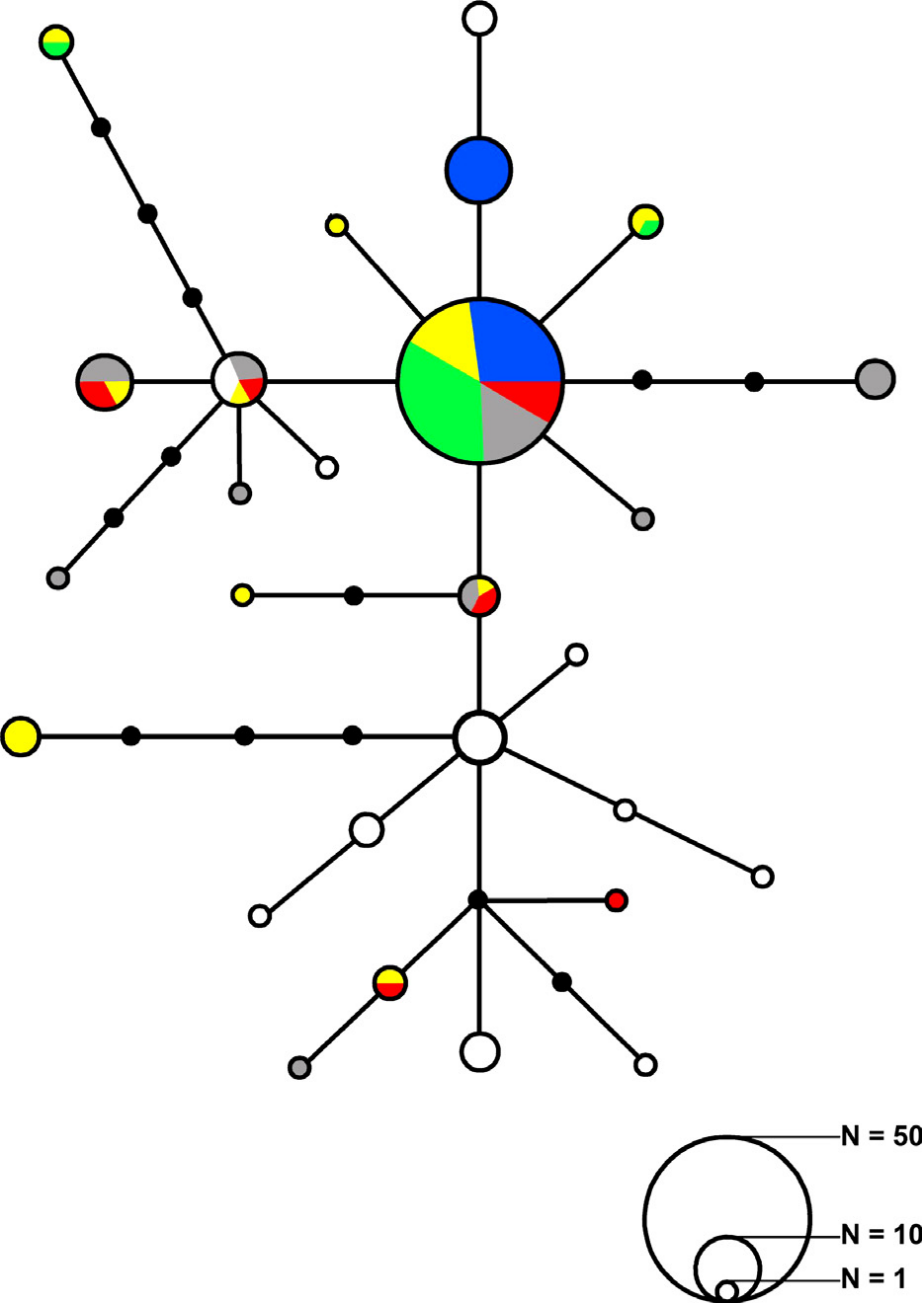
Median-joining network of mtCR haplotypes for *Mustelus henlei*. Circles represent individual haplotypes with size proportional to frequency, branches indicate mutations, and black circles are hypothetical ancestors. Localities are as follows: San Francisco Bay (Blue), Santa Barbara (Yellow), Santa Catalina Island (Green), Punta Lobos (Gray), San Felipe (Red), and Costa Rica (White). Numbers of samples per circle size are represented at bottom right.

Observed sequence divergences of *M. henlei* ranged between 0.10% and 0.60% (Table1A) and average pairwise nucleotide differences ranged between 0.48 and 4.27 among localities (Table1A). Effective female population sizes ranged between 12,586 and 179,849 (Table1A). Significant mtDNA genetic structure was observed with 34.61% (*P *<* *0.00001) of the variation being observed among sampled localities (Table2A). Pairwise Φ_ST_ values ranged between 0 and 0.51 with San Francisco and Costa Rica demonstrating significant pairwise divergences (*P *≤* *0.0033) between all other localities (Table3). Overall, migration rates determined by MIGRATE-N were asymmetric with gene flow occurring predominantly in a southward direction (Fig. 4; Appendix S2A). On regional scales, migration between Santa Catalina and Santa Barbara demonstrated asymmetric rates with Santa Barbara receiving immigrants from Santa Catalina and the three southern localities (Punta Lobos, San Felipe, and Costa Rica) demonstrating a complete lack of emigration into neighboring localities (Appendix S2A). Significant isolation by distance was observed (*r* = 0.22, *P *<* *0.00001) when all sample localities were analyzed together. When Costa Rica was excluded from the analysis, nonsignificant isolation by distance (*P *=* *0.451) was observed. The median-joining network demonstrated a general lack of divergence among localities with many of the haplotypes diverging from the most frequently observed haplotype, SB15, by a single mutation (Fig.2).

**Table 2:** Fixation indices for *Mustelus henlei*

Φ-Statistics				
Source of variation	df	Sum of squares	Variance components	Percentage of variation
(A) Φ_ST_ values for *Mustelus henlei*.				
Among populations	5	53.493	0.47231 Va	34.61
Within populations	120	107.096	0.89246 Vb	65.39
Total	125	160.589	1.36478	100
Fixation index (Φ_ST_)	0.35			

*F*-Statistics				
Source of variation	df	Sum of squares	Variance components	Percentage of variation
(B) *F*_ST_ values for *Mustelus henlei*.				
Among populations	5	66.042	0.209 Va	12
Within populations	332	527.855	0.159 Vb	88
Total	337	593.896	1.79900	100
Fixation index (*F*_ST_)	0.12			

*P *<* *0.00001.

**Table 3:** Pairwise *F*_ST_ and Φ_ST_ values for *Mustelus henlei*. *F*_ST_ values are presented below the diagonal and Φ_ST_ values above

	San Francisco	Santa Barbara	Santa Catalina	Punta Lobos	San Felipe	Costa Rica
San Francisco	–	0.19*	0.17*	0.18*	0.26*	0.51*
Santa Barbara	0.05*	–	0	0.03	0	0.26*
Santa Catalina	0.07*	0.02	–	0.08	0.19*	0.55*
Punta Lobos	0.08*	0.02	0.02*	–	0	0.33*
San Felipe	0.04	0.03	0.04*	0.03	–	0.26*
Costa Rica	0.17*	0.08*	0.13*	0.13*	0.12*	–

* indicates *P* values ≤0.0033 after sequential Bonferroni correction.

#### Female population expansion

Of the localities, Santa Barbara, Santa Catalina, Punta Lobos, and Costa Rica demonstrated negative Tajima's *D* values with significance (*P *≤* *0.05) being observed in all but Costa Rica (Table1A). Negative *F*_S_ values were observed in Santa Barbara, Punta Lobos, and Costa Rica with only Santa Barbara and Costa Rica being significant (*P *≤* *0.02) (Table1). Results of mismatch distributions were not significant for all sample localities, and therefore, the null hypothesis of recent demographic expansion could not be rejected (Table4). Tau values derived from the mismatch distributions provided estimates of coalescence in the range of 1.1–3.1 million years before present, except San Francisco (the northernmost) that was an order of magnitude more recent (Table4).

**Table 4:** Estimates of Tau, *θ*_0_, *θ*_1_, and expansion times (T) for sample localities of *Mustelus henlei*. SSD is the sum of squared differences from mismatch distributions with significant values (*P *≤* *0.05 tablewide) indicated by ^*^

Locality	Lower Bound	Mean	Upper Bound
San Francisco			
SSD = 0.019			
Tau	0	0.70	1.38
*θ*_0_	0	0	0.49
*θ*_1_	9.37	99,999	99,999
T	0	3,17,569	6,26,454
Santa Barbara			
SSD = 0.028			
Tau	0.28	6.70	12.35
*θ*_0_	0	0	1.83
*θ*_1_	1.42	4.34	99,999
T	1,27,597	30,39,575	56,04,398
Santa Catalina			
SSD = 0.013			
Tau	0.63	3.00	3.00
*θ*_0_	0	0	0
*θ*_1_	0	0.05	99,999
T	2,85,318	13,61,003	13,61,003
Punta Lobos			
SSD = 0.032			
Tau	1.10	6.90	11.65
*θ*_0_	0	0	3.00
*θ*_1_	4.67	8.89	99,999
T	4,99,742	31,30,308	52,79,825
San Felipe			
SSD = 0.024			
Tau	0.12	5.40	30.38
*θ*_0_	0	0	3.33
*θ*_1_	0.96	4.26	99,999
T	54,934	24,49,809	1,37,82,821
Costa Rica			
SSD = 0.003			
Tau	0.31	2.00	5.81
*θ*_0_	0	0.21	1.07
*θ*_1_	2.69	5.93	99,999
T	1,74,577	11,10,375	6,85,910

### Microsatellite data

#### Genetic diversity

MICRO-CHECKER detected an excess of homozygotes and the possibility of null alleles due to stuttering for Mh6, Mh13, and Mh36. However, these results were generally locality specific (heterozygote deficit in Mh6 was only observed in San Francisco, in Mh13 only in Santa Barbara, and in Mh36 only in San Francisco and Santa Barbara; Appendix S1). To determine the effect of null alleles on downstream analyses, analyses were run with and without these loci as well as with corrected frequencies estimated by MICRO-CHECKER. The following results were all observed with and without these loci as well as with corrected frequencies (with the exception of the STRUCTURE analysis described below). Observed heterozygosities (H_O_) ranged between 0.40 and 0.54 and expected heterozygosities (H_E_) were between 0.40 and 0.56 (Table1B). All loci were in HWE after corrections for multiple tests, and there was no evidence of LD for any pair of loci. Observed number of alleles within localities ranged between 22 and 35 and allelic richness ranged between 2.77 and 4.64 (Table1B). Private alleles were detected in San Francisco, Santa Barbara, and Costa Rica with Santa Barbara possessing the greatest number of alleles (Table1B).

#### Genetic divergence, population structure, and gene flow

**Figure 3:**
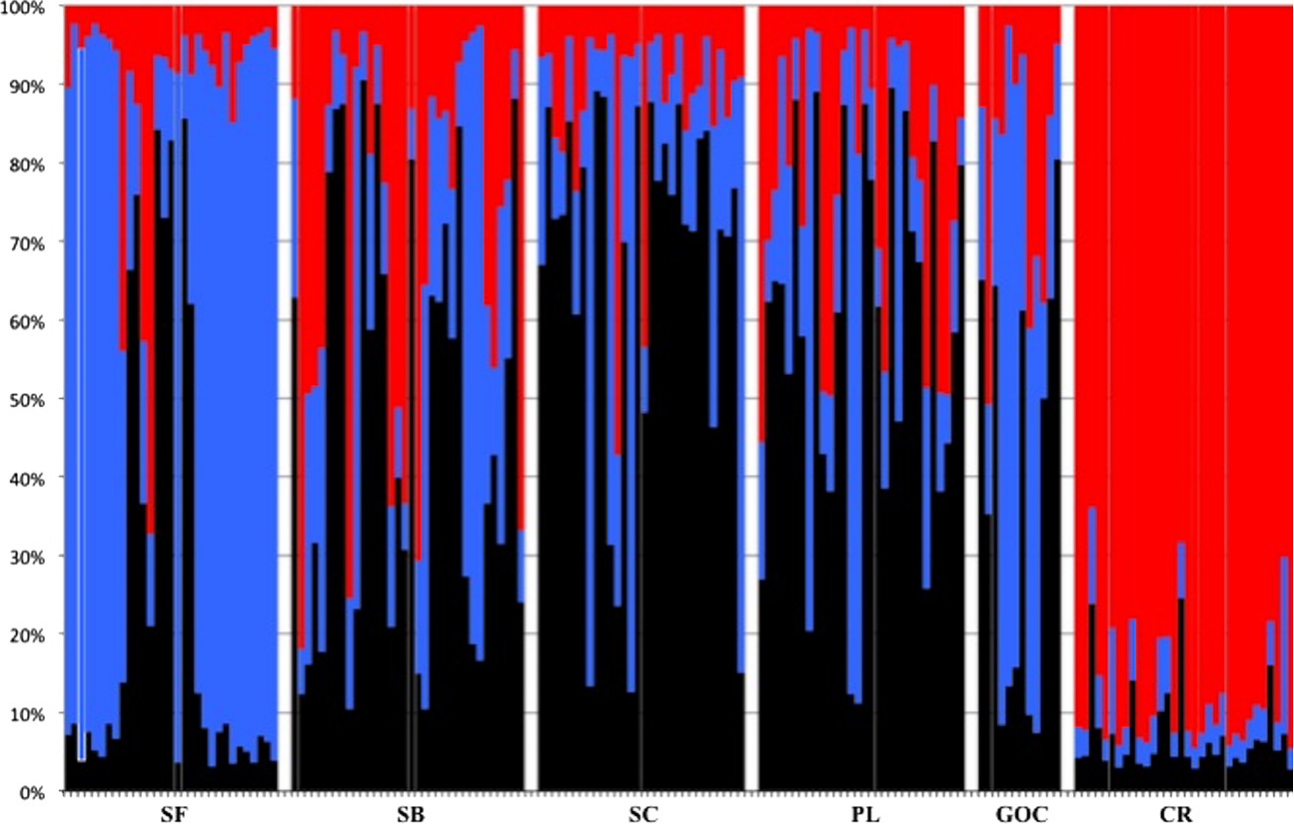
Population assignment of *Mustelus henlei* as estimated by STRUCTURE. Each individual is represented as a single histogram with percentage of ancestry on the *y*-axis and populations on the *x*-axis (SF = San Francisco, SB = Santa Barbara, SC = Santa Catalina, PL = Punta Lobos, GOC = San Felipe, and CR = Costa Rica). Spaces have been inserted between sample localities.

**Figure 4:**
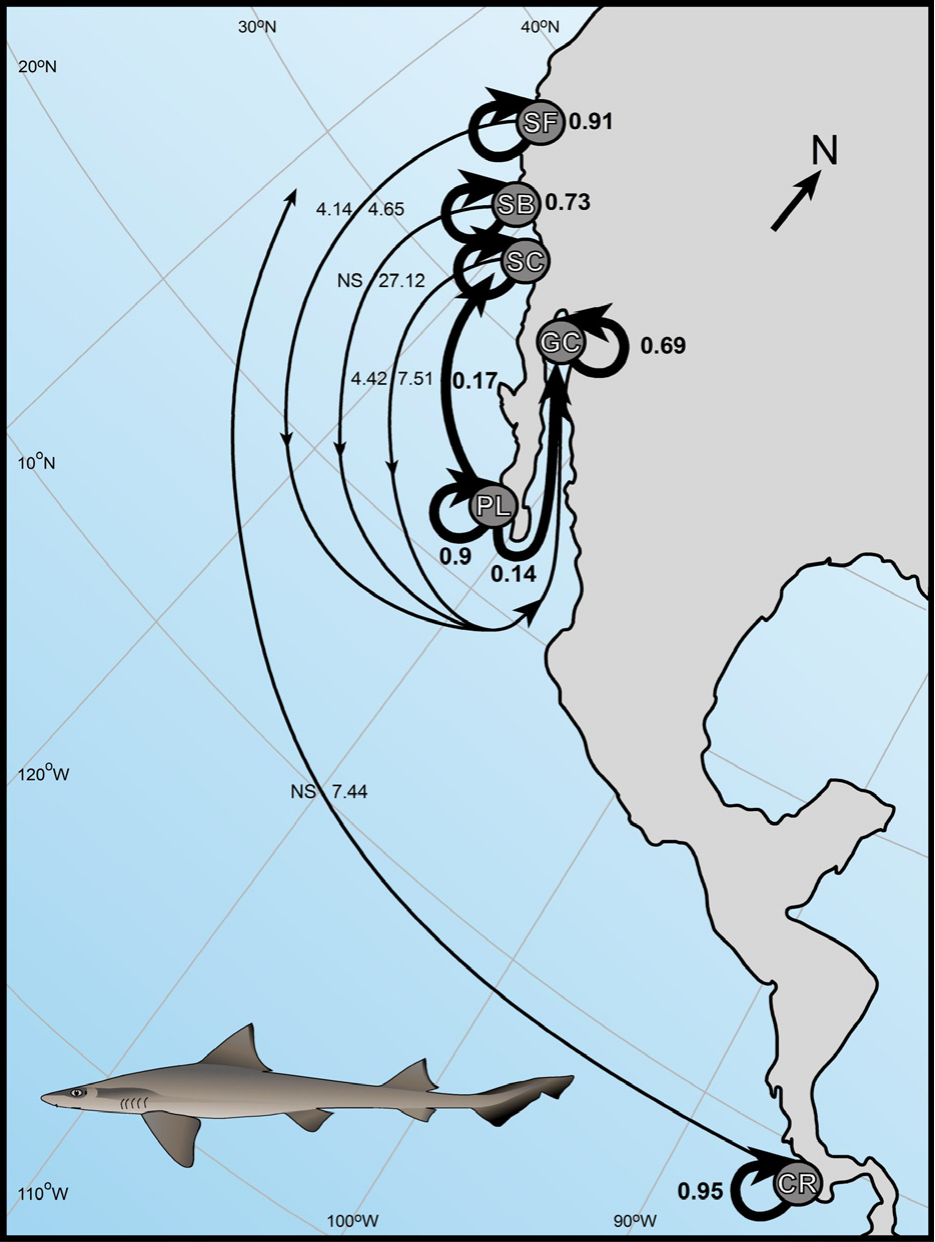
Long-term and contemporary patterns of gene flow (*N*_*e*_m) for *Mustelus henlei*. Localities are labeled as follows: San Francisco (SF), Santa Barbara (SB), Santa Catalina (SC), Punta Lobos (PB), San Felipe (GC), and Costa Rica (CR). Thin lines depict the results of MIGRATE-N analyses and the general direction of gene flow as averaged across localities from the point of origin. Estimates of *N*_*e*_m with credible sets (CS) not overlapping 0 are provided on either side of lines (mtCR to the left and microsatellites to the right). Bold lines depict the direction of gene flow as estimated by BAYESASS with *N*_*e*_m values with CS's not including 0 provided.

Results of Δ*K* from STRUCTURE HARVESTER based on the STRUCTURE analysis (Fig.3) indicated the greatest posterior probability of *K *=* *3 for all sample localities of *M. henlei* with and without the inclusion of putative null alleles. However, after correcting frequencies for null alleles, a *K = *2 demonstrated the greatest Δ*K* with San Francisco clustering with the central population. Overall, AMOVA demonstrated significant structure among sampled localities (*F*_ST_ = 0.12; *P *<* *0.00001) (Table2B). Significant genetic structure based on pairwise *F*_ST_ values was detected between San Francisco, Santa Catalina, and Costa Rica and all other localities after Bonferroni corrections, with the exception of San Felipe and Santa Barbara that were not significantly different from San Francisco and Santa Catalina, respectively (Table3). Pairwise *G′′*_ST_ and estimates of Jost's D recovered the same pattern of genetic divergence as those of pairwise *F*_ST_ presented above (Table5). Significant isolation by distance was observed (*r* = 0.21 *P *<* *0.00001) when all sample localities were analyzed together. However, when Costa Rica was excluded from the analysis, significant, yet reduced isolation by distance was observed. (*r* = 0.08, *P *=* *0.002) Long-term estimates of gene flow among population pairs were asymmetric with the predominant direction of gene flow being in a north–south direction (Fig.4; Appendix S2B). Interestingly, the opposite pattern is observed in Costa Rica as the direction of gene flow is from the south to the north (Fig.4; Appendix S2B). Overall, estimates of contemporary gene flow demonstrated a lack of significant gene flow among sample localities (Appendix S3). However, Punta Lobos did contribute significantly to Santa Barbara, Santa Catalina, and San Felipe but not to San Francisco and Costa Rica (Fig.4; Appendix S3). Of note, algorithm bounds in BAYESASS limit the proportion of nonmigrants and migrants to 0.67 and 0.33, respectively and values in these ranges may not be indicative of actual migration proportions. As the estimates of migration (0.138–0.186; Appendix S3) and 95% credible sets (0.007–0.318; Appendix S3) from Punta Lobos to Santa Barbara, Santa Catalina, and San Felipe are less than the lower model bound, these estimates are not representative of the limitations imposed by the model.

**Table 5:** Average *G*′′_ST_ and Jost's D for *Mustelus henlei. G*′′_ST_ values are presented below the diagonal and Jost's D values above

	San Francisco	Santa Barbara	Santa Catalina	Punta Lobos	San Felipe	Costa Rica
San Francisco	–	0.10*	0.15*	0.15*	0.04	0.31*
Santa Barbara	0.17*	–	0.02	0.03	0.03	0.15*
Santa Catalina	0.25*	0.03	–	0.03*	0.06*	0.26*
Punta Lobos	0.25*	0.06	0.06*	–	0.03	0.24*
San Felipe	0.08	0.05	0.11*	0.06	–	0.21*
Costa Rica	0.50*	0.27*	0.42*	0.41*	0.36*	–

* indicates *P* values ≤0.0033 after sequential Bonferroni correction.

#### Population bottleneck

BOTTLENECK did not reveal any significant heterozygote excess in any of the populations of *M. henlei* (Table1B). Garza–Williamson M ratio values were all between 0.74 and 0.83 and above critical values of M_C_ with the exception of San Francisco (Table1B).

## Discussion

### Biogeographic and phylogeographic barriers

This is the first range-wide genetic investigation of gene flow of a member of *Mustelus* in the northeastern Pacific. Significant structure was observed among sample localities of *M. henlei* based on mitochondrial control region sequences and nuclear microsatellite data (Tables2 and 3, and Fig.3). Three distinct populations can be described: a northern population made up entirely of individuals from San Francisco, a central population composed of individuals from Santa Barbara, Santa Catalina, Punta Lobos, and San Felipe, and a southern population composed entirely of individuals from Costa Rica. These populations correspond to the biogeographic provinces of the Oregonian, a blending of the San Diegan and Cortez Provinces, and the Panamic Provinces (Horn et al. 2006; Stephens et al. 2006; Robertson and Cramer 2009). Throughout these provinces, there are several biogeographic and phylogeographic barriers that have been described for various taxa with Point Conception, the Los Angeles Region (LAR), and the Peninsula of Baja California exerting the most influence on the population connectivity of northeastern Pacific temperate taxa (Rawson et al. 1999; Stepien et al. 2000; Dawson 2001; Dawson et al. 2001; Bernardi et al. 2003; Jacobs et al. 2004; Hyde and Vetter 2009).

The classification of Point Conception as a biogeographic barrier (Briggs 1974) and the LAR as a phylogeographic barrier (Dawson 2001; Dawson et al. 2001) is generally based on the patterns of observed disjunctions between populations of relatively small-sized taxa with limited adult dispersal that rely on currents to distribute larvae. However, as further studies of taxa with distributions spanning, this area have demonstrated varying degrees of disjunction across the barrier, Point Conception has been reclassified as a gradual transition zone between northern and southern lineages (Horn et al. 2006; Pelc et al. 2009; Briggs and Bowen 2013). Species with large, actively dispersing adults such as the leopard shark, *Triakis semifasciata* (Triakidae), and the California halibut, *Paralichthys californicus*, from the northeastern Pacific with distributions spanning the LAR and Point Conception have revealed similar patterns of gene flow across Point Conception but contrasting patterns across the LAR. Craig et al. (2011) used mitochondrial data to reveal an overall lack of structure among populations of *P. californicus* sampled from throughout the northeastern Pacific and a lack of structure specifically across the LAR and Point Conception and concluded that the species is comprised of a single panmictic population, at least over evolutionary time scales. Lewallen et al. (2007) observed significant structure among sample localities of *T. semifasciata* based on mitochondrial data and reduced nuclear genetic variation among sample localities between Elkhorn Slough (Monterey Bay, CA) and the LAR. Based on these observations, Lewallen et al. (2007) concluded that the LAR provided a diffuse barrier to gene flow and acted as a transition zone between populations North and South of the LAR and that Point Conception had no effect on population connectivity.

Along the California coast, *P. californicus*, *T. semifasciata*, and *M. henlei* are commonly encountered together. Commercial halibut trawlers commonly encounter *M. henlei* during trawls (M. McCorkle, pers. comm.) and leopard sharks have been observed among aggregations of *M. henlei* (Ebert 2003) and share various life-history characteristics including preferences in habitat (Love 1996) and food (Haeseker and Cech 1993; Webber and Cech 1998). Based on co-occurrence and shared life-history characteristics among the three species, it follows that *M. henlei* could also be influenced by the same barrier to dispersal (LAR but not Point Conception). Instead, our data show that the northern population of San Francisco is significantly differentiated from the central population. Hence, unlike *P. californicus* and *T. semifasciata*, Point Conception may be acting as a barrier to dispersal in *M. henlei*. In regards to the LAR, patterns of population connectivity throughout this area among localities of *M. henlei* (Table3 and Fig.3) are similar to those of Craig et al. (2011) and Lewallen et al. (2007) and indicate that this phylogeographic barrier is not effective at reducing gene flow among localities within the central population of *M. henlei*.

The Peninsula of Baja California is a tectonically active region that has undergone plate spreading, uplifting, and subduction as the Gulf of California has expanded in size over the past 3.5–12 million years (Holt et al. 2000). As a result, it has been hypothesized that ephemeral seaways have existed between the Pacific and the Gulf of California over this time period with the most recent occurring in the La Paz region during the Pleistocene (Walker 1960). This seaway would have allowed for the migration of marine species across the Peninsula, while maintaining the barrier produced by the warm surface waters of the Cape (Bernardi et al. 2003). Population disjunction has been observed in numerous species with distributions spanning both sides of the Cape including spotted sandbass, *Paralabrax maculatofasciatus*, (Tranah and Allen 1999; Stepien et al. 2000), opaleye, *Girella nigricans*, (Bernardi et al. 2003), grunion, *Leuresthis tenuis/sardina* (Bernardi et al. 2003), sargo, *Anisotremus davidsonii* (Bernardi et al. 2003), longjaw mudsucker, *Gillichthys mirabilis* (Bernardi et al. 2003), shovelnose guitarfish, *Rhinobatos productus* (Sandoval-Castillo et al. 2004), golden cownose ray, *Rhinoptera steindachneri* (Sandoval-Castillo and Rocha-Olivares 2011), and banded guitarfish, *Zapteryx exasperata* (Castillo-Páez et al. 2014). As demonstrated by the variety of species listed above, this barrier is effective at inhibiting gene flow among species with varying size, vagility, and reproductive strategies including but not limited to broadcast spawners or internal fertilizers followed by either bipartite life-history stages that utilize planktonic larval dispersal or highly vagile neonates as observed in sharks, skates, and rays. The generally accepted mechanism associated with the lack of gene flow observed between Pacific and Baja California populations at the Peninsula is the convergence of Pacific and Gulf Currents at the Cape where cold-temperate species are unable to round the Cape due to elevated sea surface temperatures (Bernardi et al. 2003). Although this mechanism may be effective at impeding gene flow in the majority of observed species, this barrier may not exert as much of an influence on taxa capable of swimming beneath warmer surface waters such as the California sheephead, *Semicossyphus pulcher*, and the round stingray, *Urobatis halleri*, species that have been collected at depths greater than 50 m within this region (Bernardi et al. 2003) and have demonstrated gene flow between the Pacific and the Gulf of California (Bernardi et al. 2003; Plank et al. 2010). Similar to *S. pulcher* and *U. halleri*, *M. henlei* is commonly collected at depths between 60 m and 200 m throughout its distribution (Ebert 2003), a range of depths that may allow the species to swim beneath the warm surface waters of the Cape and to disperse around the Cape of the Baja California Peninsula resulting in the population connectivity observed in the present study between sample localities on both sides of the Peninsula (Table3 and Fig.3).

The observation of a distinct southern population of *M. henlei* at Costa Rica in the present study corresponding to the Panamic Province may be the result of various biogeographic barriers distributed along the coast of Central America that have acted in concert to isolate populations (Robertson and Cramer 2009). Of these, the Sinaloan Gap has been associated with the southern boundary of the Cortez Province for soft-bottomed species (Robertson and Cramer 2009). Based on the affinity of *M. henlei* for soft-bottomed habitat (Compagno 1984; Castro 2011), this region may indicate a possible location as to where the disjunction between the central and southern populations occurs. Aside from the barriers described above, distance may also be responsible for the lack of connectivity between the central and southern populations of *M. henlei*. Significant isolation by distance was observed in the present study; driven primarily by the divergence between Costa Rica and all other sampled localities. As the Costa Rican population is the greatest distance from all other populations (>5000 km to the nearest localities of San Felipe and Punta Lobos), this finding is not surprising based on a tagging study of *M. henlei* in which an individual traveled a maximum distance of ∼150 km over a three-month period (Ebert 2003). Regardless of isolating mechanism, several lines of evidence including genetic, phenotypic, and ecological suggest that the southern population of *M. henlei* has most likely been isolated from northern populations for some time and may be undergoing incipient speciation. Evidence is that in fish from Costa Rica, virtually all of the mitochondrial haplotypes are unique to that population; the existence of private microsatellite alleles; a smaller TL and size at maturity when compared to *M. henlei* in higher latitudes (Clarke et al. 2014); and an observed shift in the preferred diet of the species when compared to northern and central populations (fish and cephalopods versus crustaceans, respectively) (Espinoza et al. 2012).

### Demographic expansions and bottlenecks

Historic changes in the intensity of upwelling, varying sea surface temperatures, and repeated glaciations have resulted in a climatically dynamic coastline throughout the northeastern Pacific that has affected the distributions of numerous marine taxa (Hickerson and Ross 2001; Dawson et al. 2006). One expected effect of oscillating climate cycles along the coastline is the expansion or contraction (i.e., population bottleneck) of marine populations. Expansion times for *M. henlei* based on mean Tau values support an expansion from the central population of *M. henlei* to the north and to the south (Table4). Specifically, Punta Lobos and Santa Barbara have expansion times well into the Pliocene followed by San Felipe in the Plio–Pleistocene, Santa Catalina, and Costa Rica in the early to mid-Pleistocene, and San Francisco within the mid to late Pleistocene (Table4). Demographic expansions or population bottlenecks can leave a mark on the genetic diversity of populations by either generating novel genetic variants as a result of rapidly expanding effective population sizes (Rogers and Harpending 1992) or significantly reducing genetic diversity due to severe reductions in population sizes (Cornuet and Luikart 1996; Piry et al. 1999; Garza and Williamson 2001). BOTTLENECK and M ratio tests did not detect any significant excesses in heterozygosity or reductions in the number of alleles relative to the range of allele sizes that would be indicative of bottlenecks for sample localities of *M. henlei* with the exception of San Francisco (Table1B). The value of M for San Francisco was well below the 0.69 threshold established for populations that have undergone a historic bottleneck as described by Garza and Williamson (2001). However, BOTTLENECK did not detect the signature of a bottleneck for this locality. It has been noted by Williamson-Natesan (2005) that the two methods are generally better for detecting bottlenecks over different time scales as well as under different mutation rates and prebottleneck population sizes. Generally, detecting a bottleneck using heterozygote excess was more effective when the bottleneck occurred recently, mutation rates were low, and the prebottleneck population sizes were small (Williamson-Natesan 2005). In contrast, using the range in allele size conditioned on the number of alleles worked best when bottlenecks occurred over several generations, mutation rates were high, and prebottleneck population sizes were large (Williamson-Natesan 2005). Therefore, the observed incongruence between the two methods in the sample from San Francisco would seem to indicate a historic population bottleneck that may be due to a founder's event associated with the expansion of the species’ range during periods of climate change.

Of the sampled localities, San Francisco and Santa Catalina demonstrated the lowest mtDNA diversity (Table1A) as both were composed of only two haplotypes with the majority being the most frequent haplotype observed in the present study. In San Francisco, this apparent reduction in diversity may be due to a historic range expansion northward into the area that would later become San Francisco Bay followed by the subsequent colonization of the bay after its formation ∼10,000 years before present (ybp) (Cohen 2000) at the end of the last glacial maximum. However, mean coalescent times for *M. henlei* from San Francisco are well in excess of the formation of San Francisco Bay <10,000 ybp (Table4). This apparent lack of concordance could be due to the use of a mutation rate in the present study that is not specific to the species or genus. Therefore, caution must be used when interpreting expansion times as they may be under/overestimates and are only used here to describe the magnitude of divergence among expansion estimates. Similar to the present study, a cline of genetic variation in the northeastern Pacific has also been observed in the acorn barnacle, *Balanus glandula*, by Sotka et al. (2004). Sotka et al. (2004) suggested that one explanation for the genetic pattern that they observed was that populations on both sides of the observed cline might have been isolated historically during glacial periods followed by range expansions and secondary contact once glacial ice retreated.

In regards to Santa Catalina, it is possible that during the early Pleistocene gene flow may have been relatively uninterrupted between this locality and coastal localities (e.g., Santa Barbara) due to lowered sea levels and shallower coastal basins. However, as sea level began to rise and coastal basins became deeper, population connectivity may have been reduced resulting in the more recent expansion estimate for Santa Catalina in the early to mid-Pleistocene and the observation of relatively low mitochondrial diversity (e.g., only two haplotypes). Deep-water basins have been shown to be effective barriers to population connectivity among the northern Channel Islands (Bernardi 2000) and in taxa distributed on both sides of the San Pedro Basin within the Southern California Bight (e.g., between Santa Catalina Island and the southern California coastline) (Gaida 1997; Bernardi 2000; Plank et al. 2010). However, the nonsignificant population divergence and estimates of long-term gene flow observed in the present study between Santa Catalina and Santa Barbara (Table3 and Appendix S2) do not support the role of deep-water basins as barriers to population connectivity in *M. henlei* within the Southern California Bight.

### Historic and contemporary gene flow

The North Pacific has been described as an “evolutionary engine that produces flora and fauna capable of transgressing biogeographic boundaries and become established elsewhere” (Briggs and Bowen 2013). These interhemispheric dispersals generally occur from north to south (Briggs and Bowen 2013). Extirpations during the Pleistocene may have allowed northeastern Pacific lineages to invade South American waters and establish populations within the region (Jacobs et al. 2004). Results of the MIGRATE analyses in the present study support a general pattern of long-term north to south dispersal (Fig.4; Appendix S2) that may have led to the establishment of *M. henlei* in South America. As populations of *M. henlei* are known from Peru and Ecuador (Compagno 1984; Eschmeyer et al. 1999) and this region comprises the southern limit of the species’ distribution, it would be expected that this region would have reduced genetic diversity and a more recent expansion time when compared to the populations in the North. Further investigation of this region is needed to confirm this hypothesis and elucidate the patterns of gene flow within the terminus of the southern distribution of *M. henlei*.

Contemporary migration estimates from BAYESASS would seem to paint a different picture from those based on long-term migration. Overall, a lack of significant migration was observed to localities such as San Francisco, Santa Barbara, Santa Catalina, San Felipe, and Costa Rica (Fig.4; Appendix S3). However, significant migration was observed within the central population with Punta Lobos contributing migrants to all localities (Fig.4; Appendix S3). This south to north pattern, with the exception of the Upper Gulf of California, may be indicative of the expansion of tropical *M. henlei* into subtropical and temperate waters. Although the Upper Gulf of California is separated from the temperate waters of the San Diegan Province, the Upper Gulf of California is considered to have a temperate climate as well as temperate taxa associated with California (Robertson and Cramer 2009).

### Conservation

As a family, the Triakidae has been the subject of global exploitation for greater than 80 years with members being exploited by commercial fisheries for their large vitamin-A-rich livers (e.g., *Galeorhinus galeus*), by artisanal and recreational fisheries for consumption, and by the aquarium trade (e.g., *M. henlei, M. californicus, and T. semifasciata*) (Yudin and Cailliet 1990; Ebert 2003; Compagno et al. 2005; Lewallen et al. 2007). Fishery pressure on *M. henlei* is regionally variable with interest in U.S. waters north of Mexico being minimal and the majority of landings of *M. henlei* being attributed to by-catch or by recreational anglers as a commercial fishery no longer exists in these waters (Pérez-Jimenéz and Carlisle 2009). However, in the northern waters of the Gulf of California, *M. henlei* is still of considerable commercial and artisanal importance and has been the most abundant species of shark caught during fall and winter months as landings have been in excess of 150 kg per hour (Pérez-Jimenéz and Carlisle 2009). Furthermore, fishing pressure by artisanal and semi-industrial fisheries (trawling, long-lines, and gill nets) is high in Costa Rica and there is currently no management or regulations on catch limits of *M. henlei* (M. Espinoza, pers. obs.). Currently, the IUCN Red List has listed *M. henlei* with the threat category Least Concern due to the observed lack of overfishing of the species, its fast growth-rate coupled with a relatively low longevity, an early age at first maturity with a relatively high fecundity, and a lack of evidence of catch declines (Pérez-Jimenéz and Carlisle 2009). Estimated female effective population sizes (*N*_*ef*_) for *M. henlei* observed in this study were all relatively high ranging from ∼13,000 to 180,000 (Table1A). As *N*_*ef*_ is generally considered to be ∼10% of the female census size of a population (Frankham et al. 2010), estimated census sizes of sample localities from this study would be expected to be rather large and would support the threat category assigned by the IUCN. However, the generalization of Frankham et al. (2010) is based on an average obtained from many studies with a huge range in both effective population and census sizes and may not be generalizable to sharks. Recent observations in the sandbar shark, *Carcharhinus plumbeus*, have demonstrated that the effective population size within a population can approximate the census size (Portnoy et al. 2009). Therefore, based on the observation of Portnoy et al. (2009), it is possible that the estimates of effective female population size obtained in the present study may approximate the census size of female populations of *M. henlei*. Aside from estimates of effective population size, the significant gene flow observed among localities, at least within the central population, detected in this study along with the observed genetic variation observed in Santa Barbara (greatest observed genetic diversity with several locality-specific haplotypes and private alleles) and Punta Lobos (several locality-specific haplotypes) would be expected to genetically buffer *M. henlei* from short-term population declines of small effect.

## Conclusion

As this study is the first range-wide investigation into the gene flow and population connectivity of a member of the northeastern Pacific *Mustelus*, three patterns have been observed. First, throughout the range of *M. henlei,* there exist three distinct populations with the northern (Northern California) and southern (Costa Rica) populations demonstrating significant divergence from the central population (Central-Southern California and Mexico). Second, traditional biogeographic and phylogeographic barriers within the central population appear to have little effect on the population connectivity of *M. henlei*. And third, contemporary expansion of *M. henlei* from the tropics to subtropical and temperate regions may have been revealed. As always, further sampling from different localities would be expected to bolster the results of any population genetic study of a species in the wild. With this in mind, the further sampling of *M. henlei* from Washington, Humboldt Bay, CA, Tomales Bay, CA, Morro Bay, CA, Peru, and Ecuador would seem appropriate in order to elucidate patterns of gene flow among localities within the northern and southern limits of the species’ range. Finally, when these findings are integrated with those of other nearshore, northeastern Pacific taxa, the effect of biogeographic and phylogeographic barriers on population connectivity within the region will be made far clearer and our ability to predict and manage marine resources will be greatly improved.

## Supporting Information

**Appendix S1.** Summary statistics for 6 loci from within 6 localities of *Mustelus henlei*: San Francisco (SF), Santa Barbara (SB), Santa Catalina (SC), Punta Lobos (PL), San Felipe (GOC), and Costa Rica (CR). N, number of samples; A, number of alleles; A_R_, allelic richness; H_O_, observed heterozygosity; H_E_, expected heterozygosity; *F*_IS,_ correlation of alleles within individuals compared to their subpopulation; *p*, *F*_IS_ *p* values (significant *F*_IS_ *p* values ≤ 0.00167 indicated by *). **Appendix S2**. Avergage long-term migration rates between sample localities of Mustelus henlei as estimated by MIGRATE-N. **Appendix S3.** Average **c**ontemporary migration rates (*N*_*e*_m) between sample localities of *Mustelus henlei* and self-recruitment (e.g., from donor locality San Francisco into recipient locality San Francisco) as estimated by BAYESASS. Bold values with a credible set (CS) not including 0 are provided below each pairwise population estimate and self-recruitment estimates are in the diagonal with corresponding CS values in italics below.
